# Agricultural Migrants’ Health and Ability to Access Care: A Case Study in Southern Italy

**DOI:** 10.3390/ijerph182312615

**Published:** 2021-11-30

**Authors:** Alessandro Lamberti-Castronuovo, Jeremy A. Pine, Giorgio Brogiato, Hans-Friedemann Kinkel

**Affiliations:** 1 CRIMEDIM—Center for Research and Training in Disaster Medicine, Humanitarian Aid and Global Health, Università del Piemonte Orientale, 28100 Novara, Italy; alessandro.lamberti@uniupo.it; 2 iNGO EMERGENCY, 20122 Milan, Italy; giorgio.brogiato@emergency.it; 3 Independent Scholar, 10407 Berlin, Germany; jeremypine@gmail.com; 4 Institute of Tropical Medicine and International Health, Charité–Universitätsmedizin Berlin, 13353 Berlin, Germany

**Keywords:** vulnerable, agricultural labor, barriers to access, marginalized, Italy

## Abstract

Although a large amount of research exists about migration into the European Union (EU) and the role of migrants in European society, relatively little information is available on the health status of migrants after arriving in the EU. This is particularly true in the case of the most marginalised migrants, migrants from sub-Saharan Africa, who work as itinerant laborers harvesting fruits and vegetables in southern Italy. This study analyzes demographic and health data gathered by a non-governmental organization-run primary healthcare clinic in order to understand the challenges these migrants face when trying to maintain their health. Results show that their health suffers greatly due to substandard living and working conditions, partially due to the fact that these individuals experience many barriers when trying to access care from the national health system. The health status of this population cannot improve without broad reforms to the welfare system and the agricultural sector. Government action is needed to ensure that such individuals are not denied their basic human rights and freedoms, including the right to health.

## 1. Introduction

“*African workers and all consumers who buy these products are involved in a rotten agricultural food chain where the fruits of nature are fed by the sweat and blood of illegal immigrants*”

—Antonello Mangano, 2014 [1].

“*In Southern Italy, where harvesting prevails, employment is irregular and seasonal. The foreign worker in agriculture tends to be male, young, low skilled, generally employed in areas of low specialization requiring physical strength…. Labourers are, in fact, paid on a per-piece basis, earning 3–4 euros for every 300 kg box of picked tomatoes, which implies a high work rate. Even if regular contracts are signed, a reduced number of working days is subsequently declared*”

—Alessandra Corrado, 2017 [2].

This study examines the health status of a population of migrant agricultural laborers in southern Italy and the barriers this population must overcome in order to access healthcare.

Living on the margins of society and working in a sector plagued by illegality and mafia control, this group of people faces multiple and intersecting forms of discrimination. To date, little research has been done on the specific health risks, conditions, needs, and outcomes of heavily marginalized migrant workers in Europe [3,4,5,6]. We use the term “marginalized migrant worker” to distinguish this group from other migrant workers who are a legal part of the official labor market and have access to the regular health and social protection system.

The research focuses on a shantytown/tent city settlement outside of the village of San Ferdinando in Calabria, Italy, where approximately 3000 migrants from sub-Saharan Africa reside. The part of Calabria where the settlement is located is known to be one of the poorest regions in the European Union. 

Due to the systemic lack of integration into Italian society, the group in the study experiences substandard social determinants of health (precarious and low-wage work, poor educational opportunities, racial segregation, food insecurity and inaccessibility of nutritious food choices, poor access to housing and utility services, little access to information and services in native language, etc.). This situation effectively bars settlement residents from accessing state healthcare [7]. Administrative bodies are designed to function within a framework of normative assumptions about how individuals are integrated into state services. Vulnerable populations that have fallen out of, or that have never been integrated into, this framework are rendered invisible or only selectively seen in the eyes of the state. As a result, non-governmental organizations (NGOs) and other humanitarian initiatives fill these gaps by providing care where the need is greatest. This research is based on data from the humanitarian international NGO EMERGENCY that operates a clinic in the nearby town of Polistena to serve residents of the San Ferdinando settlement. 

**Figure 1:**
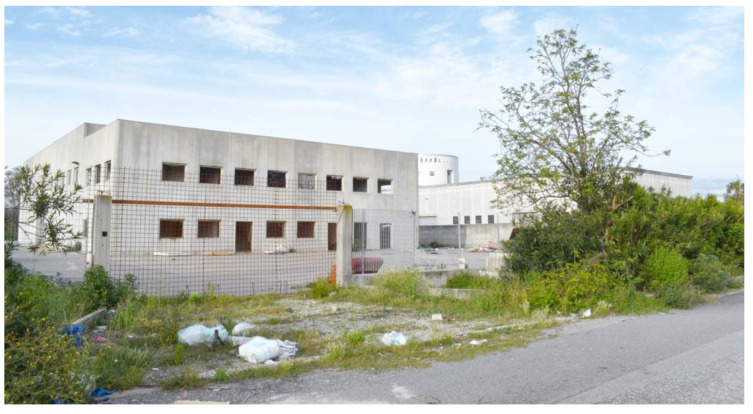
The disused industrial area abutting the San Ferdinando settlement.

The San Ferdinando settlement covers an area of approximately 500 square meters, located on the outskirts of a village of the same name (Figure 1). It is surrounded by open countryside dotted with abandoned factories and deserted farmhouses, physical representations of the area’s depressed economic circumstances. While many young Italians flee the area in order to find better opportunities in cities, the agricultural villages of Calabria have become a destination for migrants who arrive in Europe by boat via the Central Mediterranean route. The inequality demonstrated by these opposing patterns of migration is reflected in the contrast between the living conditions of Italians in the area and those of the migrants in the San Ferdinando settlement, which are extremely poor even by local standards. 

**Figure 2:**
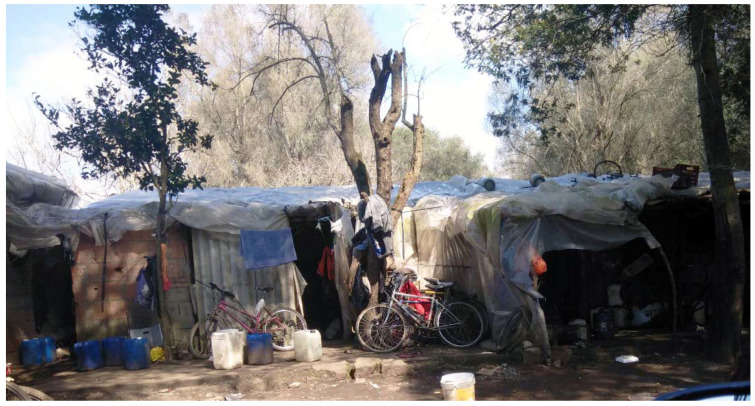
The exterior of makeshift shacks in the San Ferdinando settlement.

**Figure 3:**
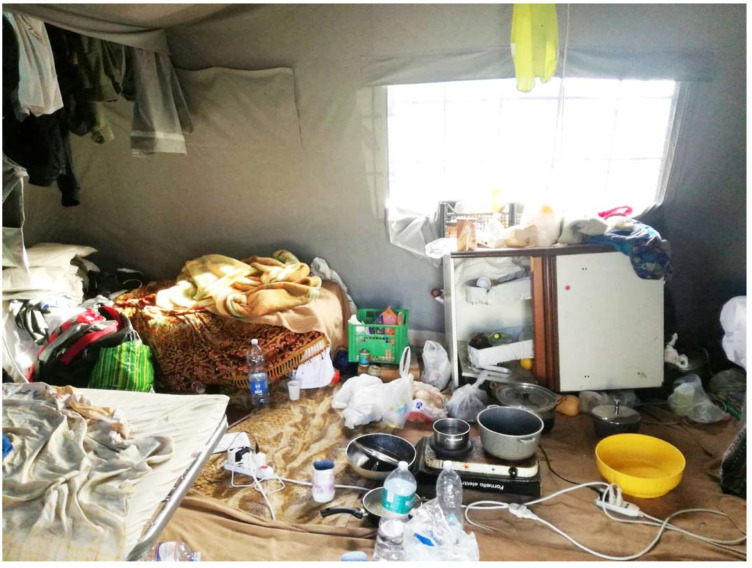
The interior of a shared tent in the San Ferdinando settlement.

The settlement comprises a tangled maze of dirt roads lined with the most modest of shelters. In July 2018, roughly 200 nylon tents along with 400 shacks cobbled together from metal and wood, covered with sheets of plastic for insulation served housing for residents (Figure 2 and Figure 3). Each of the tents housed six people, while every shack was home to 5–10 individuals. The settlement is informally arranged according to country of origin. Women residing in the settlement predominantly come from Nigeria and make up approximately 10% of the population. 

Although there is a small year round population in the San Ferdinando settlement, the majority of residents only stay there while they can find work on nearby farms. These workers move around southern Italy throughout the year in a circuit between similar settlements across the regions of Sicily, Calabria, and Puglia as different crops come into season in each of these areas. The highly mobile nature of agricultural work prevents laborers from putting down roots and building relationships within the community. It reinforces a cycle of marginalization, contributing to instability and the de facto denial of rights (e.g., rights related to residency status, access to health care, social protection, labour laws). The difficulties faced by residents are further compounded by the effects of structural racism that permeates European society and the widespread involvement of organized crime in the agricultural sector. 

**Figure 4:**
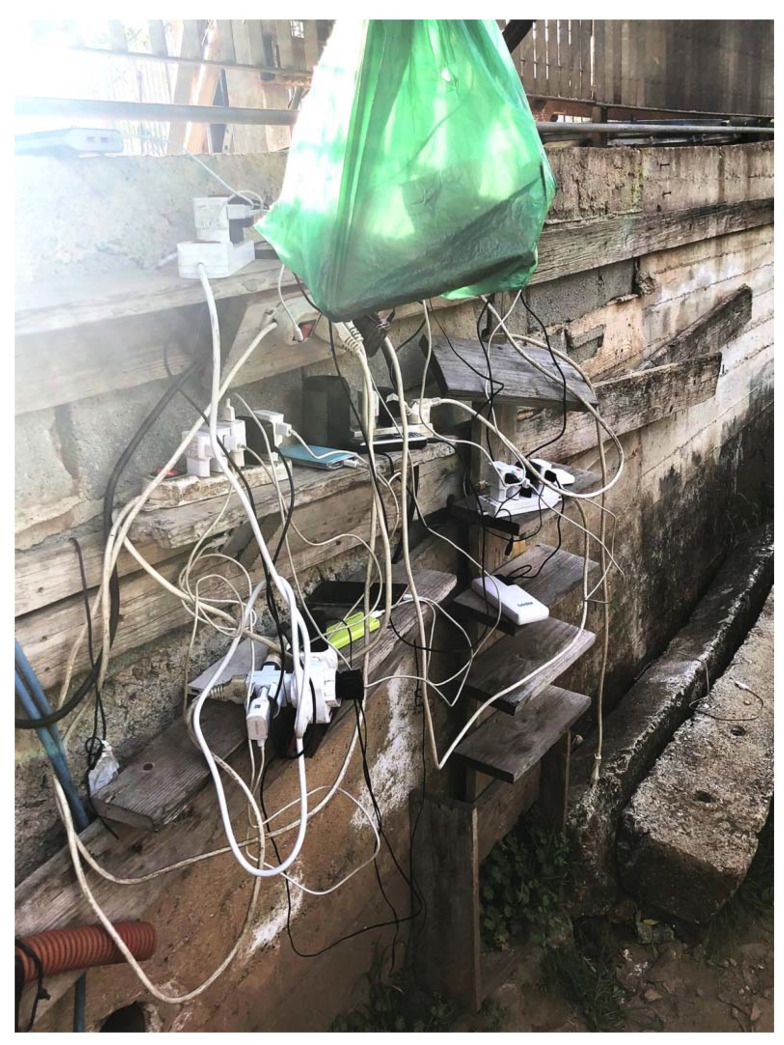
An electrical hub in a shack in the San Ferdinando settlement where residents charge their mobile phones.

The Calabrian regional government allotted the settlement area for use by migrant workers, but it has done little to make the space safe or welcoming. No running water or sewage is connected to the tents or shacks. Water is only accessible at 10 combination shower/toilet stalls. Electricity is connected to the tents and shacks via networks of extension cords, and power is only available intermittently (Figure 4). In spite of the precarious electricity delivery, there are no fire prevention measures taken within the settlement. In fact, several fires have already occurred there, resulting in the death of inhabitants [8].

Such precarious living and working conditions have an impact on the health of the settlement residents. Analyzing the data gathered by EMERGENCY, which provides basic health care services to the people of San Ferdinando, provides a rare insight into the health situation of migrant workers. 

The aim of this study is to analyze data gathered in clinical encounters with settlement residents, and to contextualize this data with insights about their living and working conditions, in order to better understand the specific health needs of this and similar populations. A systematic survey over 21,000 publications on migration health research found that: “Literature on migrant workers was very scarce (6%)… Despite their economic contributions, migrant workers, and in particular those low-skilled from lower-income nations are ‘left-behind’ in global migration health research” [9] (p. 13). A systematic review of occupational health studies found only 16 papers that focused on agricultural migrants, 14 of which were on migrant workers in the USA, one in the Gulf States and one on Thailand, but none on populations based in Europe [10]. Similarly, the 2020 World Migration Report states that research on migrant health remains limited, and primarily focused on mental health and psychosocial wellbeing [11] (p. 225). The present study fills in some gaps in the literature by providing granular health data about an under-researched group and a detailed snapshot of its health-seeking behaviors. 

## 2. Materials and Methods

This retrospective, cross-sectional study uses anonymized patient data extracted from the EMERGENCY clinic’s health information system, *wHospital*, to draw conclusions about illness/wellness and health seeking behaviors of settlement residents.

The study period was the 13 months from July 2018 to July 2019. During this time, the first author worked as a physician at the clinic, which had been established in 2010 specifically to provide free-of-charge primary health care (PHC) services to migrants in the area.

The EMERGENCY clinic collects administrative and health data about all of its patients. The administrative data includes age, gender, nationality, and legal status. The study uses administrative data to understand the demographic profile of health seekers. Health data collected by the clinic includes patients’ reasons for seeking consultation, physicians’ diagnoses according to ICD classification, medications prescribed to patients, and the distribution of medication via the clinic pharmacy. The study uses health data to assess: (1) the frequency of disease present among settlement residents seeking care at the clinic and (2) the percentage of patients who returned for mandated follow-up appointments and, by extension, the level of continuity of care. 

The clinic also collects extensive data about patients’ languages of communication (preferred language for receiving medical information, all languages spoken, language used in the medical encounter). This information is used here to calculate the percentages of patients who accessed care using (1) their preferred language, (2) a vehicular language, or (3) without sharing a common language with clinic staff, communicating as best they could with gestures and limited vocabulary. Health data is further disaggregated to show the relationship between language coverage and patients’ frequency of visits to the clinic. Language coverage’s effect on continuity of care is examined for patients diagnosed with tuberculosis. To see whether the differences found between the groups were statistically significant, the chi-squared test was used to calculate the probability value (*p*) at a confidence level of 5%. Fisher’s exact probability test was used for contingency tables with more than 20% of values of 5 or below.

The study was conducted according to the guidelines of the Declaration of Helsinki, and approved by the Ethics Committee of the A.O.U. “Maggiore della Carità” di Novara (Protocol 725/CE Study number CE 163/21) on 8 July 2021. 

## 3. Results

### 3.1. Demographics

During the study period, the clinic served 1039 patients, 598 (57.5%) of whom were residents of the San Ferdinando settlement (Table 1). These residents (*n* = 598) are the sample used for the data analysis. The mean age of the sample population was 27 years. Men made up the vast majority of the sample, accounting for 91% of individuals. All of the settlement residents served by the clinic were migrants from sub-Saharan Africa, specifically from West Africa.

Among the sample group, 128 (21.4%) were working in Italy without regular papers.

### 3.2. Burden of Disease

Of the 598 patients registered in the system, *n* = 10 patients visited the clinic for purely administrative reasons (legal aid, cultural mediation) and did not take part in a clinic encounter. Therefore, when analyzing the burden of disease in the settlement the number is *n* = 588. The most frequently diagnosed illnesses among this population were diseases of the respiratory system (35%) and of the musculoskeletal apparatus (31%). Oral health issues were the third most frequently reported diagnosis (10%) among the study population (Table 2).

Among the 49 women who presented at the clinic during the period of the study, 22 (45%) were seeking help for a gynecological/reproductive health issue. Such issues included menstrual cycle abnormalities, vaginitis, abortion counseling/services, bleeding during pregnancies, and acute conditions following self-induced abortions. 

Overall, 17 (2.9%) cases of mental health disorders were reported. Fifteen of these patients presented at the clinic with emotional or behavioral problems, symptoms of PTSD, depression, or anxiety. Notably, during the period of the study, only 2 (0.3%) patients were diagnosed with alcohol use disorder or its complications. 

HIV and tuberculosis were found at a frequency of 2 (0.3%) and 8 (1.3%) cases, respectively. Regarding non-communicable diseases, 10 (1.7%) patients were diagnosed with arterial hypertension and 12 (2%) with diabetes mellitus type 2.

### 3.3. Language Coverage and Access to Health

Only 216 patients (37%) were able to speak with clinic staff in their preferred language (PL group). Another 367 (62%) were able to communicate in a non-preferred vehicular language (VL group). Lastly, five patients (1%) shared no languages with clinic staff (NL group). 

The percentage of patients who accessed the clinic four times or more was significantly less in the VL and NL group than in the PL group (*p* < 0.0004) (Table 3).

In order to find out whether there is any correlation between language coverage and continuity of care, the same analysis was undertaken for patients with tuberculosis who were instructed to visit the clinic regularly. Although the numbers were too small to reach a level of significance, a similar trend was also seen for these patients. Of the eight patients diagnosed with tuberculosis (PL = 6, NL = 2), five patients completed their 6- or 12-month treatment plans. All five of these patients were in the PL group. Of the three who did not return for follow-up visits, one belonged to the group whose preferred language was spoken by the clinic staff, the other two could not communicate with the clinic staff in any common language. 

## 4. Discussion

There is a severe paucity of scholarly, quantitative research about foreign agricultural workers’ health status in the European Union. The majority of research that exists about this population is focused on legal protection, relying on a small number of interviews with migrants to contextualize government policies and actions [9,10,11]. However, information published in grey literature about this population corroborates the findings of the present study.

Our demographic data analysis indicates that settlement residents are young adults hailing from West Africa, the vast majority being men. A recently published report by the NGO *Medici per i diritti umani* (Doctors for Human Rights) (MEDU), which operated a mobile clinic serving San Ferdinando and comparable nearby settlements, shows similar results [12]. Over the course of five months, MEDU offered medical treatment to 484 patients, nearly all of whom came from West Africa; roughly 80% were men and the average age was 29 years. Similarly, a study of foreign workers in Italy carried out by the *Heinrich Boell Stiftung* (Heinrich Boell Foundation) (HBS) in 2017 stated that those employed in agriculture were predominantly young and male [2].

The HBS report claimed that the sub-Saharan agricultural laborers in Calabria “often have regular permits as asylum seekers or enjoy temporary international protection, or they are ‘rejected asylum seekers’, who have appealed the rejection of their asylum application and are awaiting the outcome, or they are refugees, including minors, who have evaded identification procedures.” (sic). Our results showed that about a fifth of the patients seen did not have the legal status of regular migrants to Italy, which makes it particularly difficult, if not impossible, for this group to claim any legal rights in terms of labor protection or access to health care. Moreover, even when migrants have a regular status, as is the case for nearly four fifths of the study sample, they cannot always rely on the state to guarantee their legal rights. The MEDU report, for example, shows that 90% of the patients seen in their mobile clinic are legal residents of Italy yet, merely 28% worked with legal contracts. 

Regarding the burden of disease, respiratory infections were the most common condition during medical encounters at the EMERGENCY clinic (35%). Also MEDU reported a frequency of 22.06% for respiratory infections among the population it served. The high incidence of these acute types of pathologies can very likely be explained by the crowded and unsanitary living conditions in which settlement residents find themselves and which predisposes an easy spread of airborne/droplet transmissible infections. These problems may also be exacerbated by the exposure to dust from the dry soil and plants and to pesticides during the work in agricultural fields. It was not possible to assess due to the lack of information, whether pre-existing chronic respiratory conditions or risk factors such as asthma or smoking could have contributed to the incidence of acute respiratory infections. Nonetheless, we regard the deleterious living and working conditions as the main drivers. Musculoskeletal complaints were the second most commonly reported complaints in our study population (31%). Similarly, 21% of the patients treated by the MEDU clinic presented with problems related to the osteoarticular system. These presentations can plausibly be linked to the physically demanding labor (e.g., carrying heavy loads, working in stoop or in other extreme postures, executing non-ergonomic and strenuous activities) and long working hours experienced as part of harvesting. A systematic review and meta-analysis of occupational health outcomes among international migrant workers reported that for those involved in agriculture, musculoskeletal pain and dermatological conditions were the two most common complaints [10]. 

One interesting finding from this study is the high percentage of female patients (45%) who presented with gynecological/reproductive health issues. The complaints include: menstrual cycle abnormalities, vaginitis, abortion counseling/services, bleeding during pregnancies, and acute conditions following self-induced abortions. This points to the particularly high health risks the women in our study population face. The reasons are manifold: the MEDU report, for example, states that women living in the settlements it served are likely to be victims of trafficking and/or involved in prostitution. A study conducted for the European Parliament’s Committee on Women’s Rights and Gender Equality outlines how women living in such settlements in Italy are subject to sexual exploitation [13]. Irene Peano, an anthropologist who has studied gender and migrant agricultural workers in Italy for 15 years, explains that “different forms of sexual-labour extraction have developed in relation to contemporary agro-industrial production in the Italian context along ethnicised/racialised patterns of labour composition” [14]. The preponderance of sex work, and the fact that reproductive health is notoriously hard to access in Italy, may explain the high numbers of women seeking care for gynecological and reproductive health issues [15]. A report by Human Rights Watch in 2021 reveals that “Italy is failing to fulfill its obligations to ensure women’s access to reproductive health care” [16]. Abortion is legal in Italy during the first 90 days of pregnancy for health, economic, social, or personal reasons. However, burdensome requirements as well as extensive use of “conscientious objection” by medical personnel function to deny care to women and girls, and leave them scrambling to find services within the legal time frame.

Another notable result from the analysis of medical data is the high percentage of problems diagnosed as undefined, unspecific symptoms of pain/malaise (18.4%) and the relatively low frequency of mental health diagnoses (2.8%). This may be due to the fact that clinic staff were not equipped to perform comprehensive psychological/psychiatric evaluations. However, it is also possible that some of that 18.4% were actually seeking care for underlying psychological problems. A study of immigrant health in the UK found that, “Most patients in primary care with mental health problems present with physical complaints, which can lead to under-recognition and treatment of common mental disorders. Patients with depression or anxiety sometimes focus on physical symptoms or use culture-specific bodily idioms to express distress“ [17]. The language barrier likely also contributes to such a somatization of mental health issues in contexts like the San Ferdinando settlement.

The results of the language data analysis are in line with other findings in the literature [18,19]. Those patients who were able to communicate in a preferred language were more likely to visit the clinic, and also more likely to maintain continuity of care, than those who were unable to effectively communicate with clinic staff. A study on the impact of whether nurses could offer care to asylum seekers in their native languages found that “adequate language concordance was significantly associated with higher reporting of past experience of traumatic events and of severe psychological symptoms, contrasting with much fewer referrals to psychological care when language concordance was inadequate” [18]. 

It should be noted that this study was limited to information gathered within clinical encounters with inhabitants of the camp at the EMERGENCY clinic. Thus, the overall health status of settlement residents may be different. As the target population is also itinerant, moving between several regions as the harvest season progresses, some of the data captured in this study may be incomplete or unreflective of longer-term health issues. Nevertheless, these findings can serve as a useful approximation of the pattern of disease.

## 5. Conclusions

It can be reasonably argued that many of the health issues faced by migrants in the San Ferdinando settlement are a result of residents’ poor social determinants of health. A lack of sanitary housing and sanitation exacerbates the spread of respiratory diseases. At the same time, the segregation and racism faced by settlement residents prevent them from accessing the comprehensive healthcare available to most of the Italian population. 

The inequity in health access described above can only be corrected if it goes hand in hand with safeguarding residents’ other essential rights. As long as these individuals remain victims of labor exploitation, relegated to unsanitary ghettos, the social conditions they experience will continue to endanger their health. Furthermore, in order to ensure their right to health, they need to be able to access culturally and linguistically appropriate care within the national health system.

Among the patients who accessed the EMERGENCY clinic during the study, 78.6% are in the country legally. In theory, this legal status should give them the right to use the national health system, as well as other social support and protection mechanisms. In reality, these people face multiple barriers that prevent them from making use of their rights. Because government attempts to tackle the situation have been inadequate, ad hoc efforts to provide social protection are carried out by NGOs, voluntary initiatives, and private charities. While such efforts may be able to address acute concerns of migrants, they are not equipped to solve the underlying root causes of the problem. It is the state’s duty to spearhead a comprehensive rights-based approach not only to health but also to employment and living conditions. A rights-based approach to health would involve two factors: orienting PHC to better serve the needs of marginalized people such as working migrants, and ensuring that people’s employment and living conditions, which heavily impact on their health, and access to health care are improved. 

A more responsive model of PHC provision may include the following characteristics: (a) incorporating the voices, suggestions, needs, and wants of target populations into strategies for provision of PHC, (b) a strategy of anticipatory care that involves actively reaching out to underserved groups, (c) having translation services available at all PHC locations, (d) training healthcare professionals to pay attention to the social, economic, and political factors that contribute to wellbeing. It has been documented in the literature that PHC is often the first point of entry into state services for marginalized and vulnerable populations [20,21,22]. Therefore, building more robust communication and referral systems between providers of health and social services may further improve overall health outcomes. This study can offer insights into the unmet health and social needs of sub-Saharan migrants to Europe working in the agricultural sector. 

## Figures and Tables

**Table 1:** Demographic profile of 598 patients from the San Ferdinando settlement who accessed the clinic during the study period.

Country of Origin	Number	Males	Females	Percentage (%)
Senegal	152	151	1	25.4
Mali	120	120	-	20
The Gambia	114	114	-	19
Nigeria	55	11	44	9.2
Ghana	41	39	2	6.9
Guinea	29	29	-	4.8
Guinea Bissau	23	23	-	3.9
Burkina Faso	18	18	-	3
Cote d’Ivoire	18	18	-	3
Other	28	26	2	4.7
Total	598	549	49	

**Table 2:** ICD-based burden of disease at the EMERGENCY clinic.

Diagnosis	Number	Percentage (%)
Acute respiratory infections	206	35%
Injuries/musculoskeletal pain	184	31%
Oral health	60	10%
Skin conditions	59	10%
Gastro-intestinal diseases	55	9.4%
Eye problems	35	6%
Gynecological problems	22	45% ^1^
Ear problems	19	3.2%
Mental health disorders	17	2.9%
Diabetes mellitus type 2	12	2%
Arterial hypertension	10	1.7%
Neurological disorders	8	1.4%
Urological disorders	8	1.4%
Tuberculosis	8	1.4%
HIV	2	0.3%
Undefined, unspecific	108	18.4%

^1^ 22 of the 49 female patients presented with gynecological issues.

**Table 3:** Frequency of clinic visits by patients during the study period, by language coverage.

Language Used	3 or Less Visits	4 or More Visits	Total
PL	135 (62.5%)	81 (37.5%)	216
VL	298 (81.2%)	69 (18.8%)	367
NL	5 (100%)	0 (- %)	5
Total	438 (74.5%)	150 (25.6%)	588

## Data Availability

The data presented in this study are available on request from the corresponding author.
